# Torque coordinated control of the through-the-road (TTR) 4-wheel-drive (4WD) hybrid vehicle under extreme road conditions

**DOI:** 10.1038/s41598-023-38813-3

**Published:** 2023-07-18

**Authors:** Likang Fan, Jun Wang, Meng Deng, Yiqiang Peng, Xiuchao Bao, Hongqian Wei

**Affiliations:** 1 Vehicle Measurement, Control and Safety Key Laboratory of Sichuan Province, Xihua University, Chengdu, 100089 Sichuan China; 2 Low Emission Vehicle Research Laboratory, Beijing Institute of Technology, Beijing, 100081 China; 3 Mianyang Fulin Precision Co, Ltd, Fenghuang Middle Road #37, Fucheng Disctrict, Mianyang, Sichuan China

**Keywords:** Electrical and electronic engineering, Mechanical engineering

## Abstract

Vehicular safety is of considerable significance to the intelligent development of hybrid vehicles. However, the real-time stability control or reasonable torque distribution under the extreme road conditions remain a huge challenge due to the multiple uncertain parameters and difficulties to reconcile the handling and stability performance. To address the above problems for a through-the-road (TTR) 4-wheel-drive (4WD) hybrid vehicle, this study provides a handling and stability management (HSM) approach by incorporating the offline optimization rules and on-line model predictive control (MPC). Firstly, the vehicle dynamic model with seven degrees of freedom (7-DOF) is used to offline extract torque distribution rules (Offline-ETDR), and the online MPC feedback (Online-MPCF) is utilized to compensate the extra torque requirements for the poor effect under the extreme conditions. Accordingly, the offline optimization results and online correction are fused to provide the total torque demand given the real-time road condition detection. Finally, the real vehicle test are implemented to validate the effectiveness of the proposed torque coordination strategy. In comparison to the vehicle with no torque control strategy, the proposed method significantly improves the vehicle's cornering ability while also ensuring the high stability performance.

## Introduction

Vehicular security plays a far-reaching role in the vehicle intelligence, especially for the multiple-wheel drive vehicles. For instance, the safety-critical-oriented control strategies, including the stability control, tail swing drift control as well as electronic stability program, have always been the attention of automotive researchers^1^. Specifically, extreme driving including high velocity and the fierce steering angle is the main reason for the instability of the vehicle^2^. Therefore, many methods including active suspension (AS)^3,4^, electronic stability program (ESP)^5^, dynamic cruise control^6^, active steering control (ASC)^7^, or the use of stabilizer bars^8^ have been proposed to address the above problems. Among these methods, the issue with respective of the saturation of tire lateral force is always the concern. For instance, direct yaw control (DYC)^9^ generates the additional yaw moment to adjust the longitudinal force of other wheels, enhancing the stability of the vehicle equipped with hub motors. Zhang et al.^10^ realized the stability control by combining the DYC and AFC methods, which further supplemented the required force. Mirzaei and Mirzaeinejad^11^ designed a multi-variable oriented controller to optimize the front angles. Liang et al.^12^ used the penalty function to allocate the weight of AFS and DYC to ensure the stability of 4WD vehicles during steering.These methods effectively improve the stability of the vehicle, but the coordination of these two control methods needs further discussion.

Recently, the fast control response and control feasibility have gained more attention from automotive researchers. For instance, the hierarchical control is originally proposed in^13^ and^14^ to pursue the control of speed and accuracy. In the upper DYC controller, the additional yaw moment is provided with the reference behavior; in the lower layer, multiple objectives, including the energy economy^13^, lateral stability^15,16^, the road cohesion optimization based on specific rules^17,18^ as well as the power transmission optimization^19^, are optimized to distribute the torque into four individual executors. As for the control algorithm, the fuzzy proportion-integral controllers^20,21^ were widely used to optimize the lateral force, however, the traditional PID has limited effect on vehicle stability. In references^22^ and^23^, the sliding mode control (SMC) was used to ensure the longitudinal and transverse stability of the vehicle. However, the downside of SMC is difficult to eliminate transitory buffeting. Nowadays, with the development of artificial intelligence, the optimization approaches in the traditional control strategies have been considered. Martinsen et al.^24^ used the reinforcement learning (RL) algorithm to track the reference trajectory and meanwhile guarantee the lateral stability. Wang et al.^25^ tuned the SMC parameters by the deep deterministic policy gradient algorithm in the RL optimization. Similarly, in^26^, the RL scheme was used to tune PID controller parameters. Wei et al.^27,28^ combined with vehicle safety and energy utilization efficiency, a torque coordination control strategy based deep RL was proposed, subsequently, the effectiveness of this strategy was proved by the simulation. However, the RL approach is not suitable for the online operation due to its increased computational overhead. Thus, a more feasible method, named mode predictive control (MPC)^29–33^, has been considered for the real-time execution and multiple constraints. The literature^34^ integrated the AFS, differential braking (DB) and 4WD into MPC for its compatibility, and the effectiveness of the strategy was demonstrated through the joint simulation of MATLAB and CarSim. Although the MPC method is widely used to optimize the lateral stability performance, the required yaw moment is still generated from the reference sideslip angle (*β*) and yaw rate (*ω*). Therefore, How to balance these two factors is important for the yaw stability control and thorough torque coordinated management, particularly in the case of extreme circumstances like the high speed or low adhesion.

The yaw moment calculated by the top controller is allocated to each wheel in accordance with the specific requirements of bottom controller. For instance, Ding et al.^17^ designed the average allocation algorithm based on the dynamic vertical loads. On this basis, Refs.^35^ and^36^ achieved a better control on the minimum tire slip criteria. In order to balance the total longitudinal force, lateral force, and yaw moment for the steering and braking stability, the weighted pseudo-inverse control method was proposed in Ref.^37^. In general, the bottom torque allocation algorithm based on optimization can achieve better control effect than the rule-based allocation algorithm, however, the existing optimization methods has consider little about the extreme road conditions and the computation overhead also requires more attentions.

As indicated, this goal of this paper is that the stability and real-time executability be taken into account when controlling the torque. Although current studies suggest that the *β* and *ω* could be regarded as the reference to the vehicle stability, there is limited information analyzing the influence mechanism of them on the vehicle handling and stability. Typically, the optimization objective function is defined based on their basic weights, neglecting the differences in their impacts on handling stability. Besides, the influence of multiple environmental factors and vehicle parameters, including vehicle velocity, road adhesion coefficients, on vehicle stability should also be considered. Ultimately, it is meaningful to form a control strategy that can be applied online.

To this end, this article develops an optimal torque coordination control strategy with considering the vehicle handling stability by combing an off-line prior state switch and an on-line model optimization. Explicitly, (1) A 7-DOF numerical model are firstly developed using the recorded data from real vehicles, which has been validated with the mutual benchmarking. (2) The torque distribution rules for various road adhesion coefficients, vehicle velocity as well as the front wheel angles are extracted offline by using the optimization technique. According to the optimization results, the effective areas using offline control rules are divided. (3) The widely used MPC approach is employed to improve the vehicle stability in those places where the offline control rules fall short. Finally, using a modified fuel powered vehicle with hybrid drive, an offline and online vehicle handling and stability control method is verified.

The following is the organizational structure of the entire article. "Design of the TTR model" section creates and validates the TTR model. “Method interpretation” section suggests the torque distribution approach, which includes the offline extraction and online correction operations. "Simulation and experimental verification" section demonstrates the efficacy of strategy through simulation and experimentation. "Conclusion" section draws the conclusion of the full text.

## Design of the TTR model

**Figure 1:**
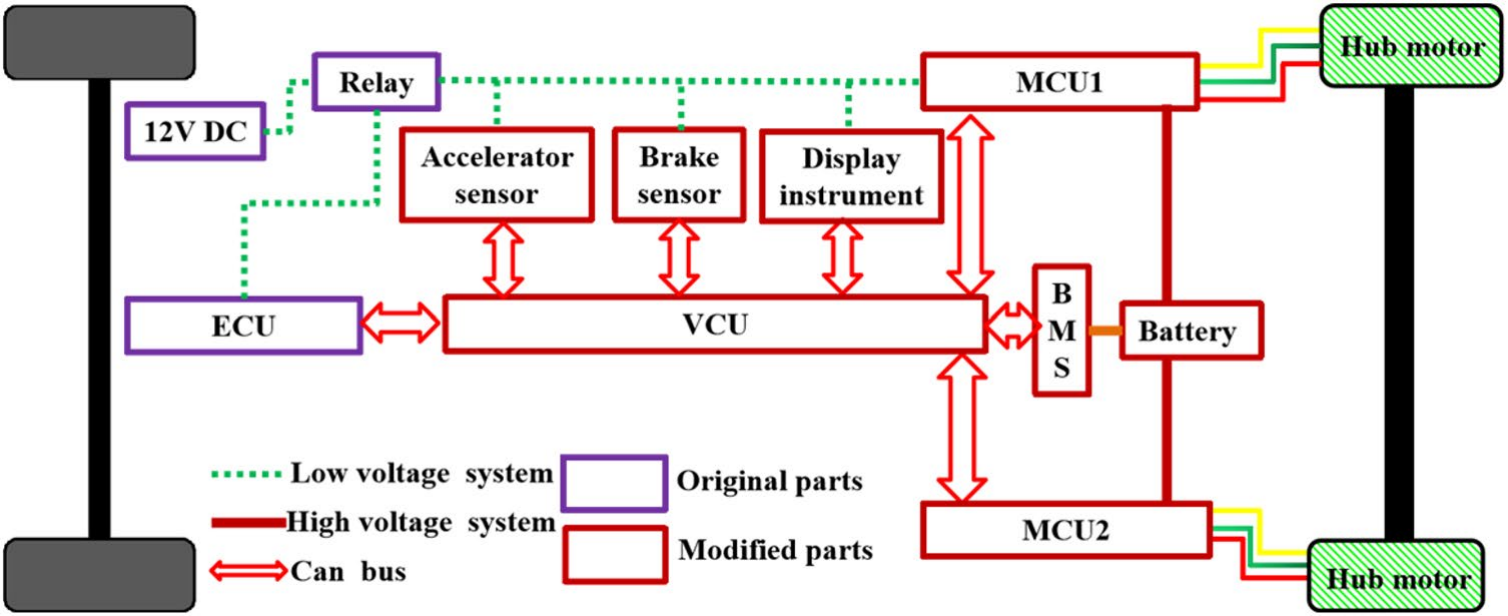
Configuration of the TTR powertrain.

The objective in this article is a four-wheel driven hybrid vehicles powered by a conventional fuel engine and two hub motors and its structure is illustrated in Fig. 1. The rear wheels are driven by two hub motors while the front wheels are still powered by the engine. The structure has discarded the traditional central differential and the transmission shaft running through the whole vehicle. In other word, four wheels are coupled through the road (TTR) friction. The TTR vehicle has the following modes including the pure engine mode, pure electric mode, driving power generation mode and hybrid drive mode. In the pure electric mode, the rear axle hub motors drive the whole vehicle to run normally. In the pure engine mode, the rear axle hub motor does not work. In the hybrid mode, the hub motors of rear axle and the engine in front axle distribute the torque requirements in real time according to the driver's intention and current road conditions, so as to ensure the fuel economy and yaw stability.

### Vehicle model

**Figure 2:**
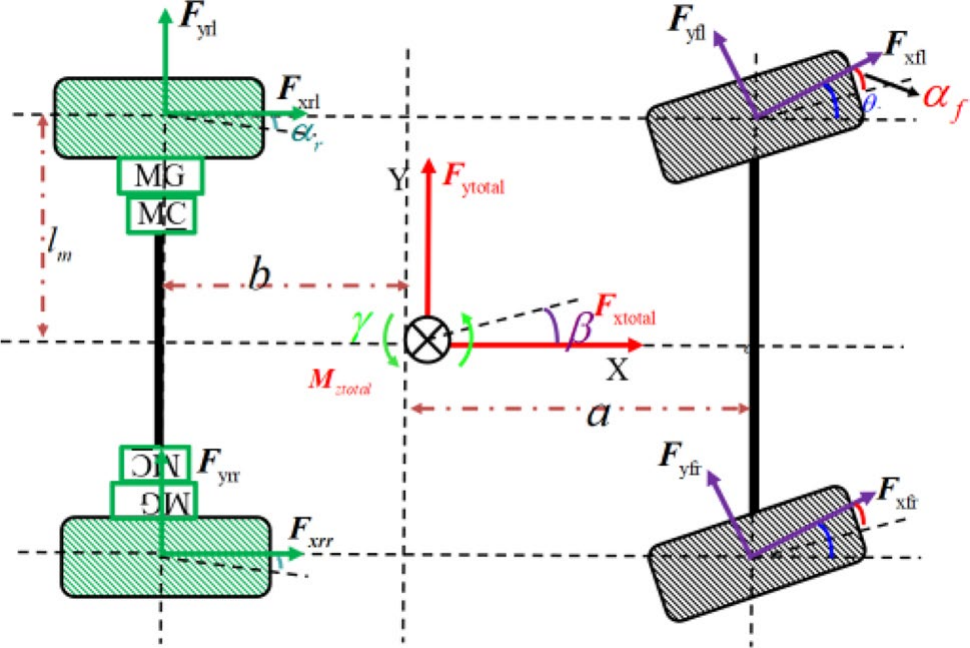
7-DOF vehicle model.

The 7-DoF model, as shown in Fig. 2, take into account the rotation of four wheels, as well as the motions of vehicle in the longitudinal, lateral, and yaw directions, to depict the performance throughout the corner steering process. At the same time, this article also makes some reasonable assumptions about the model as follows:

1. Neglecting the influence of the steering system and directly using the front wheel angle as the control input.
2. Neglecting the role of the suspension system, assuming that the carriage only moves in a plane parallel to the ground and ignoring the vertical motion of the vehicle.

Explicitly, the longitudinal dynamic equation is depicted as follows.

1 $$F_{x} = \left( {F_{xfl} + F_{xfr} } \right)\cos \theta + \left( {F_{xrl} + F_{xrr} } \right) - \left( {F_{yfl} + F_{yfr} } \right)\sin \theta - C_{D} Av_{{}}^{2} /21.15$$

where *F*_*xfl*_ and *F*_*xfr*_ are the longitudinal forces of the left and right front wheels respectively, *F*_*xrl*_ and *F*_*xrr*_ are the longitudinal forces of the left and right rear wheels respectively, *F*_*yfl*_ and *F*_*yfr*_ are the lateral forces of the left and right front wheel respectively, *θ* is the front wheel angle, *v* is the longitudinal speed, *C*_*D*_ is the air resistance coefficient, *A* is the windward area.

The lateral dynamic equation is expressed as follows.

2 $$F_{y} = \left( {F_{xfl} + F_{xfr} } \right)\sin \theta + \left( {F_{yfl} + F_{yfr} } \right)\cos \theta + \left( {F_{yrl} + F_{yrr} } \right)$$

where *F*_*yfl*_ and *F*_*yfr*_ are the lateral forces of the left and right front wheel respectively, *F*_*yrl*_ and *F*_*yrr*_ are the lateral forces of the left and right rear wheel respectively.

The yaw dynamic equation is depicted in the following.

3 $$M_{z} = \Delta M_{z} + \left( {F_{yfl} + F_{yfr} } \right)a\cos \theta - \left( {F_{yrl} + F_{yrr} } \right)b + \left( {F_{yfl} - F_{yfr} } \right)l_{m} \sin \theta$$

where *∆M*_*z*_ is the additional yaw moment, *a* is the distance from the front axle to the center of mass, *b* is the distance from the rear axle to the center of mass, and *l*_*m*_ is half the track width.

4 $$\Delta M_{z} = \left( {F_{xfl} + F_{xfr} } \right)a\sin \theta + \left( {F_{xrr} + F_{xfr} \cos \theta - F_{xfl} \cos \theta - F_{xrl} } \right)l_{m}$$

### Transmission system model

Equations (5), (6) can describe the function of the transmission system including the gearbox and clutch model numerically. Because this article focuses on analyzing the safety of the vehicle under extreme road conditions, the clutch engagement process and shift process are reasonably ignored.

The driving traction torque of the whole vehicle (*T*_*d*_) can be obtained from Eq. (5).

5 $$Td = F_{x} \times r$$

Due to the particularity of the TTR designed in this paper, the output torque of the engine (*T*_*e*_) and motors (*Tm*_*r*_*,Tm*_*l*_) is calculated according to the Eq. (6).

6 $$\frac{Te}{{i_{0} \times i_{g} \times \eta }} + Tm_{r} + Tm_{l} = Td$$

where*, T*_*d*_ is the traction torque of the whole vehicle, *r* is the tire radius, *T*_*e*_* is* the torque of the engine, *Tm*_*r*_ and*Tm*_*l*_ are the torque of the right and left hub motors, $\eta$ is the transmission efficiency, *i*_*0*_ is the speed ratio of the main reducer and *i*_*g*_ is the speed ratio of each gear of the gearbox respectively.

The transmission ratio *i*_*g*_ is shown in Table 1.

**Table 1:** Transmission ratio of the AMT and final drive.

Gear	1	2	3	4	5	Final drive
Ratio	3.28	1.92	1.26	0.87	0.72	4.06

### Engine and motor model

**Figure 3:**
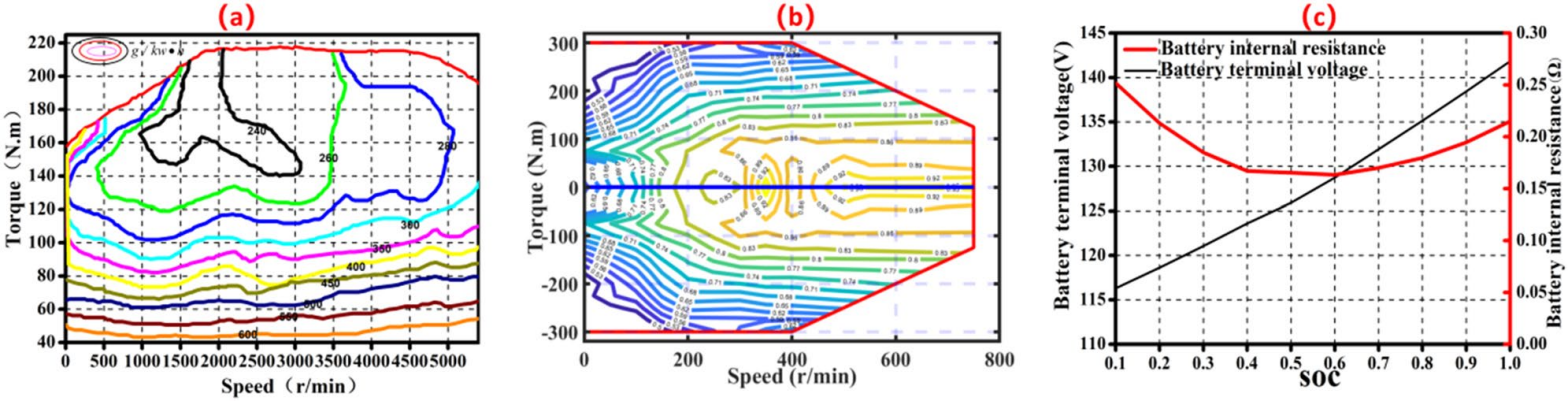
(**a**) Characteristic curve of the engine. (**b**) Characteristic curve of the motor. (**c**) The measured terminal voltage and internal resistance of single cell.

As depicted in Fig. 3a,b the engine and motor model are numerically constructed using the steady-state maps and offline experimental data. The impact of engine and motor temperature on working performance is not taken into account based on plausible assumptions.

### Battery model

In the experimental test, a Panasonic NCR-18650 circular lithium battery with 3.6 V and 3.3 A h is taken as the unit battery in this investigation. Figure 3c shows the observed terminal voltage and internal resistance of a set of cells as a function of the battery SOC.

### Tire model

**Figure 4:**
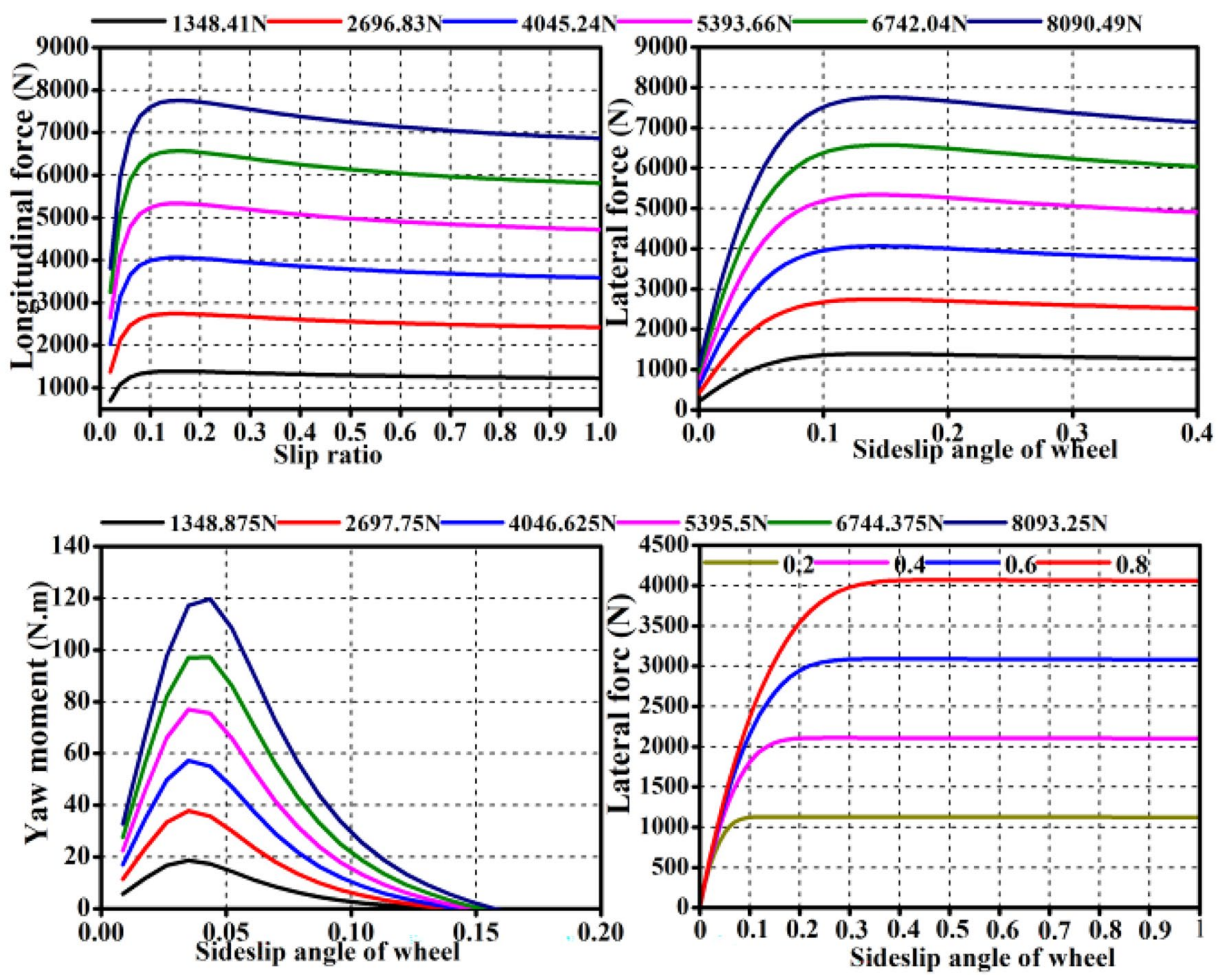
Tire model.

The calculation of lateral force in Eq. (2) is closely related to tire characteristics, the tire data of the real vehicle to be modified in this paper is 205/55 R16 from CarSim is used for modeling, and the tire characteristics are shown in Fig. 4.

It can be seen from Fig. 4 that the tire lateral force is nonlinear with the tire sideslip angle (*α*_*f*_). Therefore, this paper uses the method of looking up the table to calculate the vehicle lateral force, and the calculation of the tire sideslip angle is shown in the Eq. (7).

7 $$\left\{ {\begin{array}{*{20}l} {\alpha_{fl} = \arctan \left( {\frac{{v_{y} + \omega a}}{{v_{x} - \omega l_{m} }}} \right) - \theta } \hfill \\ {\alpha_{fr} = \arctan \left( {\frac{{v_{y} + \omega a}}{{v_{x} + \omega l_{m} }}} \right) - \theta } \hfill \\ {\alpha_{rl} = \arctan \left( {\frac{{v_{y} - \omega b}}{{v_{x} - \omega l_{m} }}} \right)} \hfill \\ {\alpha_{rr} = \arctan \left( {\frac{{v_{y} - \omega b}}{{v_{x} + \omega l_{m} }}} \right)} \hfill \\ \end{array} } \right.$$

where *α*_*fl*_*, α*_*fr*_*, α*_*rl*_ and *α*_*rr*_ are the sideslip angles of the left front wheel, the right front wheel, the left rear wheel and the right rear wheel, respectively, *ω* the yaw rate, *v*_*x*_ and *v*_*y*_ represent longitudinal and lateral velocity respectively.

### Vehicle model and verification

The Simulink 7-DOF model is then tested against a car model made in CarSim under step θ = 0.05 rad, μ = 0.6, v = 40 km/h and average torque distribution between the front and rear axles and left and right wheels. Table 2 displays the structural parameters for vehicle modeling. The results of two separate simulation programs are shown in Fig. 7.

**Table 2:** Vehicle parameters.

Name	Value	Unit
Mass	2000	kg
Vehicle width	1.49	m
Front wheel moment of inertia	0.4892	kg m^2^
Rear wheel moment of inertia	15	kg m^2^
Vehicle moment of inertia	2700	kg m^2^
Front wheel steering ratio	10:1	

**Figure 5:**
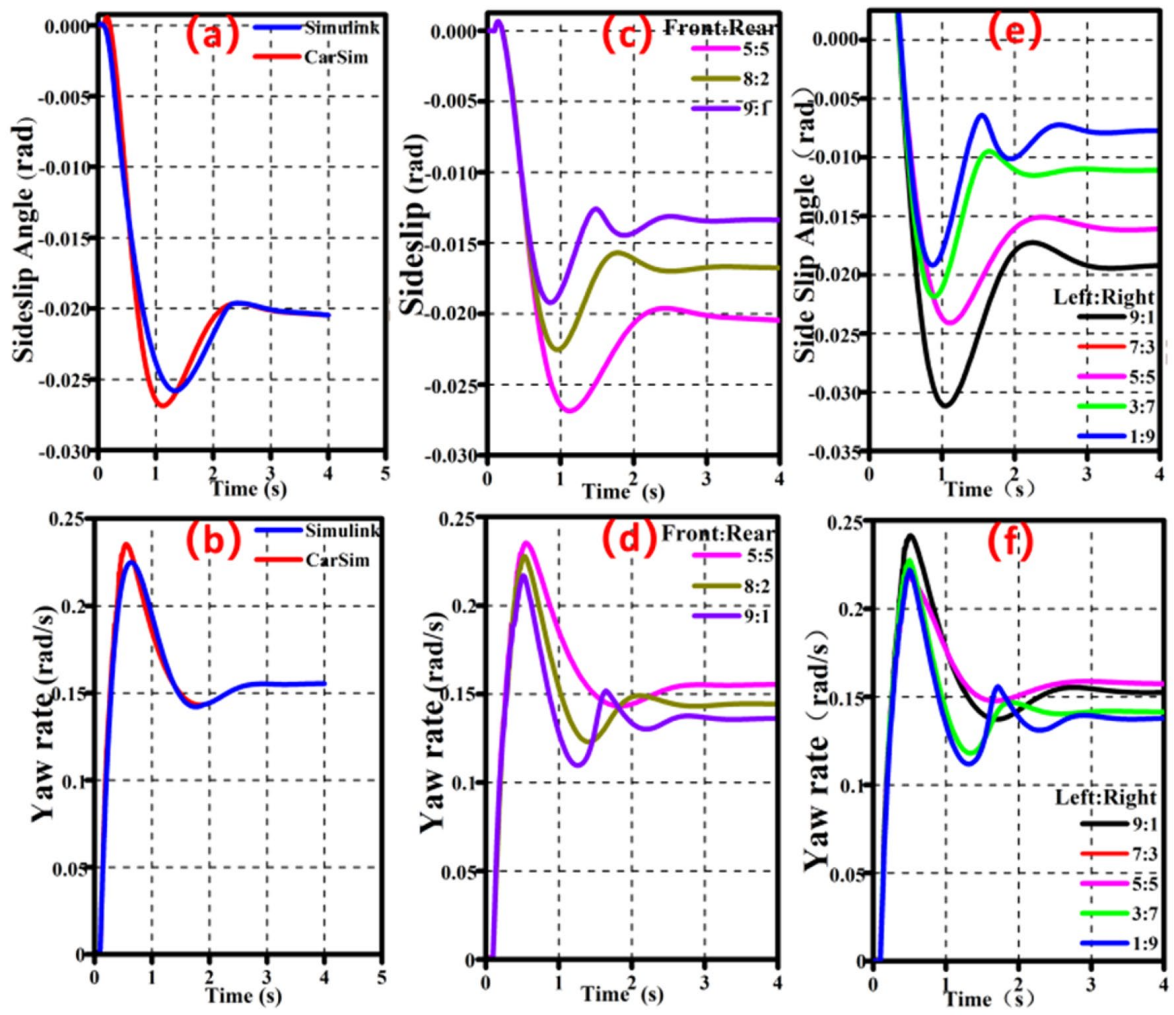
(**a**,**b**).Comparison results of different models. (**c**,**d**). Influence of front and rear axle torque on handling stability. (**e**,**f**) Influence of left and right axle torque on dandling stability.

As shown in Fig. 5a,b, the simulation outcomes of the 7-DOF model and the CarSim model are marginally different. This is due to the Simulink model disregard for the AS whereas the CarSim model accounts for additional degrees of freedom. However, based on the results of the simulation, the Simulink 7-DOF can faithfully represent movement under real-world driving circumstances because the overall inaccuracy is less than 5%. As a result, the Simulink 7-DOF forms the foundation of the remainder of this essay.

## Method interpretation

Table 3 shows the simulation setup conditions for Fig. 5c–f. Figure 5c,d shows that as the torque distribution ratio of the front and rear axles increases, the peak of *β* decreases from − 0.0257 to − 0.018 rad/s, and the peak of *ω* decreases from 0.23 to 0.22 rad/s. This shows that when the vehicle turns, the front axle obtains more power and has a certain effect on improving the excessive steering of the vehicle.

**Table 3:** Simulation setting conditions.

	Simulation setting conditions				
	Speed (*v*), km/h	Adhesion coefficient (*μ*)	Front wheel angle (*θ*), rad	Front: rear torque distribution	Left: right torque distribution
Fig. 5 c,d	80	0.8	0.05	–	5:5
Fig. 5 e,f	80	0.8	0.05	5:5	–
Fig. 6	40	0.6	0.03	The offline-ETDR	
Fig. 7 a	80	0.6	0.05	The offline-ETDR	
Fig. 7 b	40	0.2	0.03	The offline-ETDR	

Figure 5e,f illustrate that when the outer wheel reaches a 90% torque distribution compared to the inner wheel, the maximum value of *β* increases from − 0.017 to − 0.032 rad. Similarly, the maximum value of *ω* increases from 0.22 to 0.24 rad/s. It illustrates that outer wheel power transfer may boost steering capacity while inner wheel power transfer can control the vehicle's excessive steering trend.

Figure 5 shows that the magnitude of *β* and *ω* changes during steering differ substantially, and *β* remains constant for the whole vehicle in most driving scenarios. As a result, *ω* is prioritized as the optimization goal in this study.

### Extraction of the off-line control rules

Figure 5 shows that the magnitude of yaw rate and sideslip changes during the steering process vary greatly, and sideslip stays around 0 rad in most driving conditions. Therefore, simple weighting of both variables cannot measure handling stability effectively. As yaw rate reflects the turning process's severity, and sideslip indicates whether the car follows the expected turning path, this study uses yaw rate as the top optimization objective.

The offline extraction of torque distribution rules is shown in Fig. 8. The particular technique is to change the torque distribution of the front and rear axles, as well as the left and right axles, in the 7-DOF model such that the actual *ω* value always matches the reference value acquired by the 2-DOF model.

The offline control rules obtained from the simulation test are shown in Tables 4, 5, 6, 7, 8. Using the optimization results of *θ* = 0.05 rad, *μ* = 0.6, and *v* = 40 km/h as an example, 0.4/0.2 means that the front axle's driving torque accounts for 40% of the total driving torque, and the left wheel's driving torque accounts for 20% of the rear axle's driving torque.

**Table 4:** Off line control rule with front wheel angle of 0.05 rad.

Speed\coefficient of adhesion	0.2	0.4	0.6	0.8
20 km/h	0.2/0.4	0.2/0.2	0.2/0.2	0.6/0.8
40 km/h	0.8/0.6	0.6/0.8	0.4/0.2	0.4/0.2
60 km/h	0.8/0.8	0.8/0.4	0.6/0.6	0.8/0.4
80 km/h	0.8/0.8	0.2/0.8	0.6/0.8	0.8/0.6

**Table 5:** Off line control rule with front wheel angle of 0.04 rad.

Speed\coefficient of adhesion	0.2	0.4	0.6	0.8
20 km/h	0.2/0.8	0.2/0.2	0.2/0.2	0.6/0.8
40 km/h	0.8/0.2	0.4/0.2	0.4/0.4	0.8/0.8
60 km/h	0.8/0.8	0.2/0.4	0.6/0.8	0.8/0.8
80 km/h	0.8/0.8	0.2/0.8	0.2/0.8	0.8/0.8

**Table 6:** Off line control rule with front wheel angle of 0.03 rad.

Speed\coefficient of adhesion	0.2	0.4	0.6	0.8
20 km/h	0.2/0.2	0.2/0.2	0.2/0.2	0.6/0.2
40 km/h	0.2/0.8	0.2/0.2	0.8/0.4	0.6/0.2
60 km/h	0.6/0.6	0.4/0.4	0.4/0.8	0.6/0.8
80 km/h	0.8/0.8	0.8/0.2	0.2/0.8	0.8/0.2

**Table 7:** Off line control rule with front wheel angle of 0.02 rad.

Speed\coefficient of adhesion	0.2	0.4	0.6	0.8
20 km/h	0.2/0.8	0.6/0.2	0.2/0.2	0.6/0.2
40 km/h	0.2/0.6	0.6/0.2	0.6/0.8	0.6/0.2
60 km/h	0.6/0.6	0.4/0.2	0.4/0.4	0.8/0.2
80 km/h	0.8/0.8	0.6/0.8	0.6/0.8	0.6/0.6

**Table 8:** Off line control rule with front wheel angle of 0.01 rad.

Speed\coefficient of adhesion	0.2	0.4	0.6	0.8
20 km/h	0.2/0.8	0.6/0.2	0.6/0.2	0.6/0.2
40 km/h	0.4/0.4	0.6/0.8	0.6/0.8	0.6/0.6
60 km/h	0.2/0.6	0.4/0.2	0.8/0.8	0.8/0.8
80 km/h	0.4/0.8	0.4/0.8	0.6/0.2	0.8/0.2

**Figure 6:**
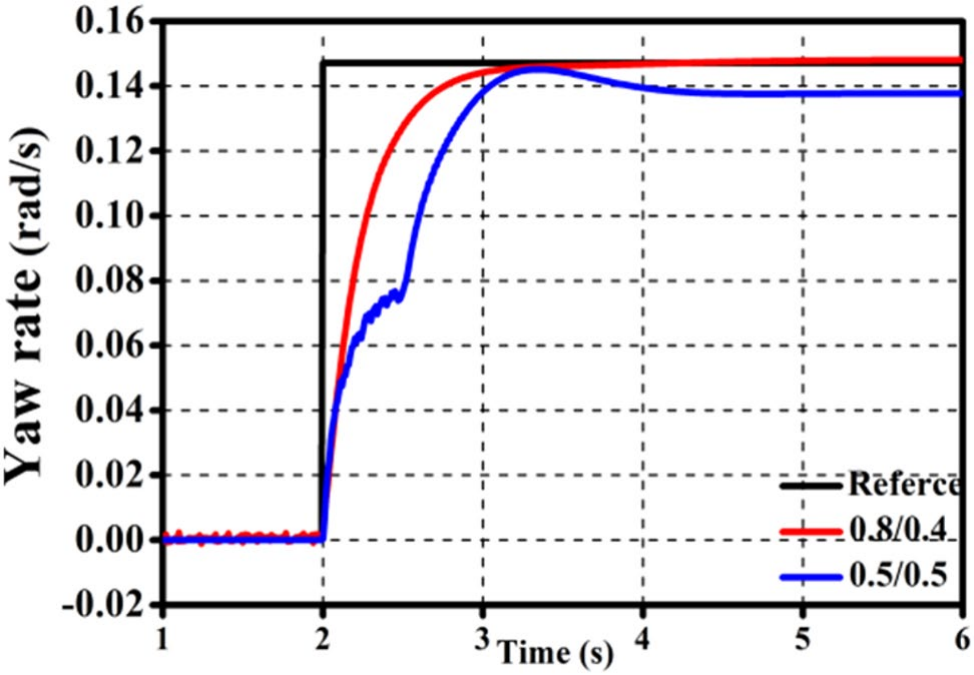
Validation of the offline-ETDR.

**Figure 7:**
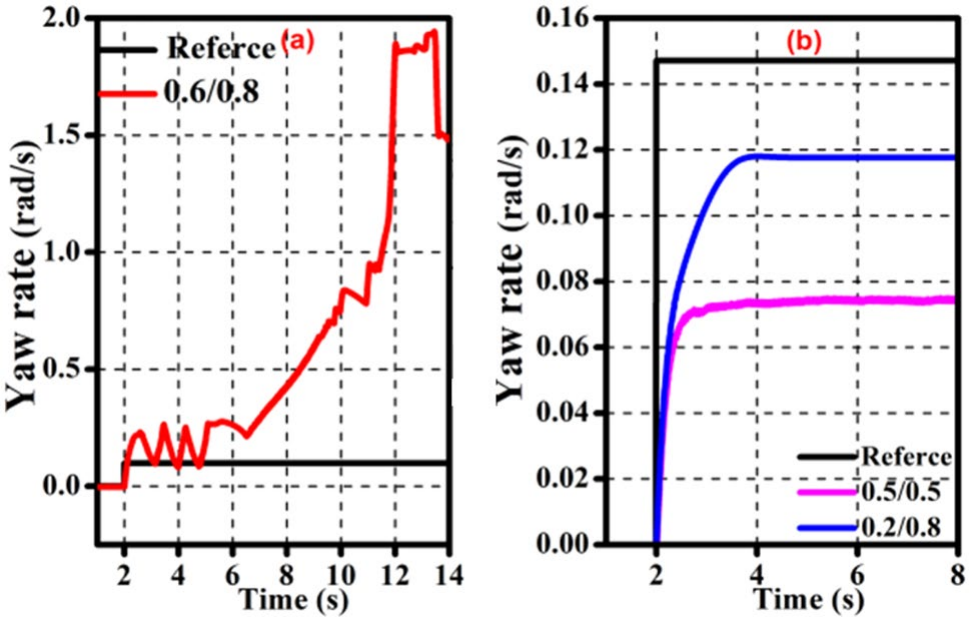
Validation of the offline-ETDR for extreme operating conditions.

The effectiveness of the off-line control rule in this study is demonstrated in Fig. 6 by the fact that the *ω* utilizing the off-line control rules (0.8/0.4) is closer to the reference. However, it can be seen from the Fig. 7a that under extreme road conditions, even if the off-line control rule is used, the whole vehicle cannot achieve the best motion state. Figure 7b shows that, even though the off-line control rule (0.2/0.8) cannot make the entire vehicle approach the reference, it is closer to it than without the optimized torque distribution ratio (0.5/0.5), demonstrating the correctness and effectiveness of the off-line control rule.

The full motion state of the vehicle cannot, as was previously indicated, be completely guaranteed by the offline-ETDR to match the reference states particularly in light of the terrible road conditions. As a consequence, the efficacy of the offline-ETDR are counted in this study, as shown in Table 9. To make the motion state of the entire vehicle match the reference state, the offline-ETDR can be used in region A. The offline-ETDR in region D has no effect on making the vehicle motion state conform to the reference state. The offline-ETDR have some control influence on region B and C, but it cannot fully verify that the actual motion state of the vehicle conforms to the reference state.

**Table 9:** The efficacy of the offline-ETDR.

Discrimination threshold	*v* ≤ 50 km/h *μ* ≥ 0.4 *θ* ≤ 0.03 rad	*v* ≤ 50 km/h *μ* ≥ 0.4 *θ* ≥ 0.03 rad	*v* ≤ 50 km/h *μ* ≤ 0.4 *θ* ≤ 0.03 rad	*v* ≤ 50 km/h *μ* ≤ 0.4 *θ* ≥ 0.03 rad
Divided area	A		B	
Discrimination threshold	*v* ≥ 50 km/h *μ* ≥ 0.4 *θ* ≤ 0.03 rad	*v* ≥ 50 km/h *μ* ≥ 0.4 *θ* ≥ 0.03 rad	*v* ≥ 50 km/h *μ* ≤ 0.4 *θ* ≤ 0.03 rad	*v* ≥ 50 km/h *μ* ≤ 0.4 *θ* ≥ 0.03 rad
Divided area	C		D	

Therefore, in regions B, C, and D, where the control effect is low, the control requirements for stability cannot be provided only by fixed torque distribution. On the one hand, the torque distribution must be flexibly adjusted according to the status of the vehicle; on the other hand, the *β* must be considered in terms of handling stability. In order to bring the motion state of the entire vehicle closer to the ideal circumstances, real-time on-line corrective control has been implemented on the basis of off-line optimization.

### Online MPC correction strategy

Since only the offline torque distribution rules are inclined to be limited on optimizing the yaw stability performance especially under extreme road conditions, the online correction process is significantly required. The model predictive control (MPC) which can foresee changes in system state caused by changes in control variables is a model-based control approach capable of controlling a dynamic system while adhering to various constraints. Thus, the real-time MPC is utilized to correct the yaw stability control trajectory.

In this study, the state variables, control variables and output variables of the control system are shown in the following.

8 $$\left\{ {\begin{array}{*{20}l} {x = \left[ {v_{x} ,v_{y} ,\omega } \right]^{T} } \hfill \\ {u = \left[ {F_{x} ,\Delta M_{z} } \right]^{T} } \hfill \\ {y = \left[ {v_{x} ,\beta ,\omega } \right]^{T} } \hfill \\ \end{array} } \right.$$

where *x* represents the state variable, *u* represents the control variable, *y* represents the output variable, *F*_*x*_ is the longitudinal traction force, *∆M*_*Z*_ is the additional yaw moment, *β* is the sideslip angle, *ω* is the yaw rate, the calculation of *F*_*x*_ and *∆M*_*Z*_ are shown in the Eqs. (1, 2, 3, 4). 

The output variable values of the system are depicted as follows.

9 $$y = \left[ {\begin{array}{*{20}c} 1 & 0 & 0 \\ 0 & {1/v_{x} } & 0 \\ 0 & 0 & 1 \\ \end{array} } \right] \cdot x$$

The state space of the system can be obtained by Eq. (10).

10 $$\dot{x} = f(x,u) = \left[ {\begin{array}{*{20}l} {\frac{1}{m}\left[ {\left( {F_{xfl} + F_{xfr} } \right)cos\theta - \left( {F_{yfl} + F_{yfr} } \right)sin\theta + F_{xrl} + F_{xrr} } \right] + v_{y} w} \hfill \\ {\frac{1}{m}\left[ {\left( {F_{xfl} + F_{xfr} } \right)sin\theta + \left( {F_{yfl} + F_{yfr} } \right)cos\theta + F_{yrl} + F_{yrr} } \right] - v_{x} w} \hfill \\ {\frac{a}{{I_{z} }}\left[ {\left( {F_{xfl} + F_{xfr} } \right)sin\theta + \left( {F_{yfl} + F_{yfr} } \right)cos\theta } \right] - \frac{b}{{I_{z} }}\left( {F_{yrl} + F_{yrr} } \right)} \hfill \\ { + \frac{{l_{m} }}{{I_{z} }}\left[ {\left( {F_{xfr} - F_{xfl} } \right)cos\theta + \left( {F_{yfl} - F_{yfr} } \right)sin\theta + \left( {F_{xrr} - F_{xrl} } \right)} \right]} \hfill \\ \end{array} } \right]$$

Due to the nonlinear characteristics of Eq. (10), Taylor formula is used for linear approximation at the reference point (*x*_*f*,_*u*_*f*_), as shown in Eq. (11).

11 $$\dot{x} \approx f(x_{r} ,u_{r} ) + \frac{\partial f}{{\partial x}}(x - x_{r} ) + \frac{\partial f}{{\partial u}}(u - u_{r} )$$

Among

12 $$\dot{x}_{r} = f(x_{r} ,u_{r} )$$

Further, Eq. (12) is subtracted from Eq. (11).

13 $$\dot{x} - \dot{x}_{r} = \frac{\partial f}{{\partial x}}(x - x_{r} ) + \frac{\partial f}{{\partial u}}(u - u_{r} )$$

To further simplify Eq. (13), the following symbols are used:

14 $$\dot{\tilde{\varsigma }} = \dot{x} - \dot{x}_{r} \, \tilde{\chi } \, = x - x_{r} \, \tilde{\psi } = \, u - u_{r} \, A = \frac{\partial f}{{\partial x}} \, B = \frac{\partial f}{{\partial u}}$$

Therefore, the linearization of the nonlinear Eq. (10) is complete.

15 $$\dot{\tilde{\varsigma }} = A\tilde{\chi } + B\tilde{\psi }$$

where the matrix* A* and* B* can be expressed as follows.

16 $$A = \frac{\partial f}{{\partial x}} = \left[ {\begin{array}{*{20}c} {\frac{{\partial f_{1} }}{{\partial x_{1} }}} & {\frac{{\partial f_{1} }}{{\partial x_{2} }}} & {\frac{{\partial f_{1} }}{{\partial x_{3} }}} \\ {\frac{{\partial f_{2} }}{{\partial x_{1} }}} & {\frac{{\partial f_{2} }}{{\partial x_{2} }}} & {\frac{{\partial f_{2} }}{{\partial x_{3} }}} \\ {\frac{{\partial f_{3} }}{{\partial x_{1} }}} & {\frac{{\partial f_{3} }}{{\partial x_{2} }}} & {\frac{{\partial f_{3} }}{{\partial x_{3} }}} \\ \end{array} } \right] = \left[ {\begin{array}{*{20}c} 0 & \omega & 0 \\ { - \omega } & 0 & 0 \\ 0 & 0 & 0 \\ \end{array} } \right]$$

17 $$B = \frac{\partial f}{{\partial u}} = \left[ {\begin{array}{*{20}l} {\frac{{\partial f_{1} }}{{\partial u_{1} }}} \hfill & {\frac{{\partial f_{2} }}{{\partial u_{2} }}} \hfill \\ {\frac{{\partial f_{1} }}{{\partial u_{1} }}} \hfill & {\frac{{\partial f_{2} }}{{\partial u_{2} }}} \hfill \\ {\frac{{\partial f_{1} }}{{\partial u_{1} }}} \hfill & {\frac{{\partial f_{2} }}{{\partial u_{2} }}} \hfill \\ \end{array} } \right] = \left[ {\begin{array}{*{20}c} {1/m} & 0 \\ 0 & 0 \\ 0 & {1/I_{z} } \\ \end{array} } \right]$$

In order to make Eq. (15) discrete, the forward Euler method as shown in Eq. (18) is used.

18 $$\dot{\tilde{\varsigma }} = \frac{{\tilde{\varsigma }\left( {k + 1} \right) - \tilde{\varsigma }\left( k \right)}}{T} = A\tilde{\chi }(k) + B\tilde{\psi }(k)$$

19 $$\tilde{\varsigma }(k + 1) = \left( {I + TA} \right)\tilde{\chi }(k) + \left( {TB} \right)\tilde{\psi }(k)$$

where *T* is the sampling time.

Further, in order to simplify Eq. (19), the following symbols are used.

20 $$\tilde{A} = I + TA \, \tilde{B} = TB$$

21 $$\tilde{\varsigma }(k + 1) = \tilde{A}\tilde{\chi }(k) + \tilde{B}\tilde{\psi }(k)$$

where

22 $$\tilde{A} = \left[ {\begin{array}{*{20}c} 1 & \omega & 0 \\ { - \omega } & 1 & 0 \\ 0 & 0 & 1 \\ \end{array} } \right]$$

23 $$\tilde{B} = \left[ {\begin{array}{*{20}l} {T/m} \hfill & 0 \hfill \\ 0 \hfill & 0 \hfill \\ 0 \hfill & {T/I_{Z} } \hfill \\ \end{array} } \right]$$

Assuming that the system prediction time domain step is *p* and the control time domain step is *m* (*m* ≤ *p*), the output variables and control variables in step* p* are expressed.

24 $$\begin{aligned} & \tilde{\varsigma }(k + 1) = \tilde{A}\tilde{\xi }(k) + \tilde{B}\tilde{u}(k) \hfill \\ & \tilde{\varsigma }(k + 2) = \tilde{A}^{2} \tilde{\xi }(k) + \tilde{A}\tilde{B}\tilde{u}(k) + \tilde{B}\tilde{u}(k + 1) \hfill \\ & \tilde{\varsigma }(k + 3) = \tilde{A}^{3} \tilde{\xi }(k) + \tilde{A}^{2} \tilde{B}\tilde{u}(k) + \tilde{A}\tilde{B}\tilde{u}(k + 1) + \tilde{B}\tilde{u}(k + 2) \hfill \\ & \, \vdots \hfill \\ & \tilde{\varsigma }(k + p) = \tilde{A}^{p} \tilde{\xi }(k) + \tilde{A}^{p - 1} \tilde{B}\tilde{u}(k) + \tilde{A}^{p - 2} \tilde{B}\tilde{u}(k + 1) + \ldots \tilde{A}^{p - m} \tilde{B}\tilde{u}(k + m - 1) \hfill \\ \end{aligned}$$

Further, in order to simplify Eq. (24), the following symbols are used.

25 $$Y = \left[ {\begin{array}{*{20}l} {\tilde{\varsigma }(k + 1)} \hfill \\ {\tilde{\varsigma }(k + 2)} \hfill \\ {\tilde{\varsigma }(k + 3)} \hfill \\ { \, \vdots } \hfill \\ {\tilde{\varsigma }(k + p)} \hfill \\ \end{array} } \right]$$

26 $$\vartheta = \left[ {\begin{array}{*{20}l} {\tilde{A}} \hfill \\ {\tilde{A}^{2} } \hfill \\ {\tilde{A}^{3} } \hfill \\ { \, \vdots } \hfill \\ \begin{gathered} \tilde{A}^{p - 1} \hfill \\ \tilde{A}^{p} \hfill \\ \end{gathered} \hfill \\ \end{array} } \right]$$

27 $$\Phi = \left[ {\begin{array}{*{20}l} {\tilde{B}} \hfill & 0 \hfill & 0 \hfill & \cdots \hfill & 0 \hfill \\ {\tilde{A}\tilde{B}} \hfill & {\tilde{B}} \hfill & {} \hfill & {} \hfill & {} \hfill \\ {\tilde{A}^{2} \tilde{B}} \hfill & {\tilde{A}\tilde{B}} \hfill & {\tilde{B}} \hfill & {} \hfill & {} \hfill \\ { \, \vdots } \hfill & {} \hfill & {} \hfill & {} \hfill & {} \hfill \\ {\tilde{A}^{p - 1} \tilde{B}} \hfill & {\tilde{A}^{p - 2} \tilde{B}} \hfill & {\tilde{A}^{p - 3} \tilde{B}} \hfill & \ldots \hfill & {\tilde{A}^{p - m} \tilde{B}} \hfill \\ \end{array} } \right]$$

28 $$\Delta U = \left[ {\begin{array}{*{20}c} {\tilde{u}(k)} \\ {\tilde{u}(k + 1)} \\ {\tilde{u}(k + 2)} \\ \vdots \\ {\tilde{u}(k + m - 1)} \\ \end{array} } \right]$$

29 $$Y = \vartheta \tilde{\xi }(k) + \Phi \Delta U$$

In order to enable the actual output to quickly track the expected value, the sideslip angle and yaw rate of the 2-DOF vehicle reference model are taken as the reference value, and its sequence is as follows.

30 $$R_{d} (k + 1) = \left[ {\begin{array}{*{20}l} {r(k + 1)} \hfill & {r(k + 2)} \hfill & \cdots \hfill & {r(k + p)} \hfill \\ \end{array} } \right]^{T}$$

In this chapter, the optimization objective as shown in Eq. (30) is written as quadratic. In addition, the weight coefficient and relaxation factor as shown in Eq. (31) are added to the optimization objective since the system model is time-varying and cannot ensure that the optimization goal can provide a workable solution at all times.

31 $$J(k) = \sum\limits_{i = 1}^{p} {\left\| {Y(k + 1) - R_{d} (k + 1)} \right\|_{Q}^{2} } + \sum\limits_{i = 1}^{m - 1} {\left( {\Delta u(k + 1)} \right)_{R}^{2} } + \rho \varepsilon^{2}$$

32 $$\sum\limits_{i = 1}^{p} {\left\| {Y(k + 1) - R_{d} (k + 1)} \right\|_{Q}^{2} } { = }\sum\limits_{i = 1}^{p} {\left[ {\left( {\omega (k + i{|}k) - \omega_{r} (k)} \right)^{2} Q_{1} + \left( {\beta (k + i{|}k) - \beta_{r} (k)} \right)^{2} Q_{2} } \right]}$$

In Eq. (32), the first term of the formula is used to punish the deviation between the predicted output and the reference in the time domain, the second term is the system's requirement for steady change, and the third term prevents the system from having no feasible solution in the control cycle.

Given the lowest tire adhesion usage rate, the longitudinal force and extra yaw moment calculated by the MPC above are fairly distributed to the front and rear axles, as well as the left and right wheels. Therefore, the torque distribution optimization problem can be expressed as the following Eq. (33).

33 $$\left\{ {\begin{array}{*{20}l} {\min J = \min \sum\limits_{i = 1}^{4} {\frac{{\lambda_{i} (F_{xfl}^{{2}} + F_{{xf{\text{r}}}}^{{2}} + F_{xrl}^{{2}} + F_{xrr}^{{2}} )}}{{\left( {\mu_{i} F_{zi} } \right)^{2} }}} } \hfill \\ {{\text{ s}}{\text{.t}}{. }\left\{ {\begin{array}{*{20}l} \begin{gathered} F_{xfl} + F_{xfr} + F_{xrl} + F_{xrr} = F_{x} \hfill \\ F_{xfl} \ge 0;F_{xfr} \ge 0;F_{xrl} \ge 0;F_{xrr} \ge 0 \hfill \\ \end{gathered} \hfill \\ {l_{m} \left( {F_{xfr} - F_{xfl} } \right) + l_{m} \left( {F_{xrr} - F_{xrl} } \right) = \Delta M_{z} } \hfill \\ \begin{gathered} \left| {F_{xfi} } \right| \le \min \left| {\left( {\mu_{i} F_{zi} ,T_{max} /r} \right)} \right|;i = r;l \hfill \\ \left| {F_{xri} } \right| \le \min \left| {\left( {\mu_{i} F_{zi} ,T_{\max } /r} \right)} \right|;i = r;l \hfill \\ \end{gathered} \hfill \\ \end{array} } \right.} \hfill \\ \end{array} } \right.$$

where *λ*_*i*_ is a constant, *F*_*zi*_ is the vertical force of each wheel, *r* is the rolling radius of the wheel,*μ*_*i*_ is the road adhesion coefficient at each tire, *∆M*_*z*_ is the additional yaw moment, *T* is the driving torque of each wheel.

The calculation of vehicle vertical force (*F*_*zi*_) in Eq. (33) can be obtained from the following Eq. (34).

34 $$\left\{ \begin{gathered} F_{zfl} = \frac{mgb}{{2L}} - \frac{{ma_{x} h_{g} }}{2L} - \frac{{ma_{y} h_{g} }}{L}\frac{b}{{l_{m} }} \hfill \\ F_{zfr} = \frac{mgb}{{2L}} - \frac{{ma_{x} h_{g} }}{2L} + \frac{{ma_{y} h_{g} }}{L}\frac{b}{{l_{m} }} \hfill \\ F_{zrl} = \frac{mga}{{2L}} + \frac{{ma_{x} h_{g} }}{2L} - \frac{{ma_{y} h_{g} }}{L}\frac{a}{{l_{m} }} \hfill \\ F_{zrr} = \frac{mga}{{2L}} + \frac{{ma_{x} h_{g} }}{2L} + \frac{{ma_{y} h_{g} }}{L}\frac{a}{{l_{m} }} \hfill \\ \end{gathered} \right.$$

where, *F*_*zfl*_, *F*_*zfr*_, *F*_*zrl*_ and *F*_*zrr*_ are the vertical loads of the left front wheel, the right front wheel, the left rear wheel and the right rear wheel, respectively, and* L* is the track width, and *h*_*g*_ is the distance from the center of mass to the ground.

### Design of off-line and on-line handling stability control strategy

**Figure 8:**
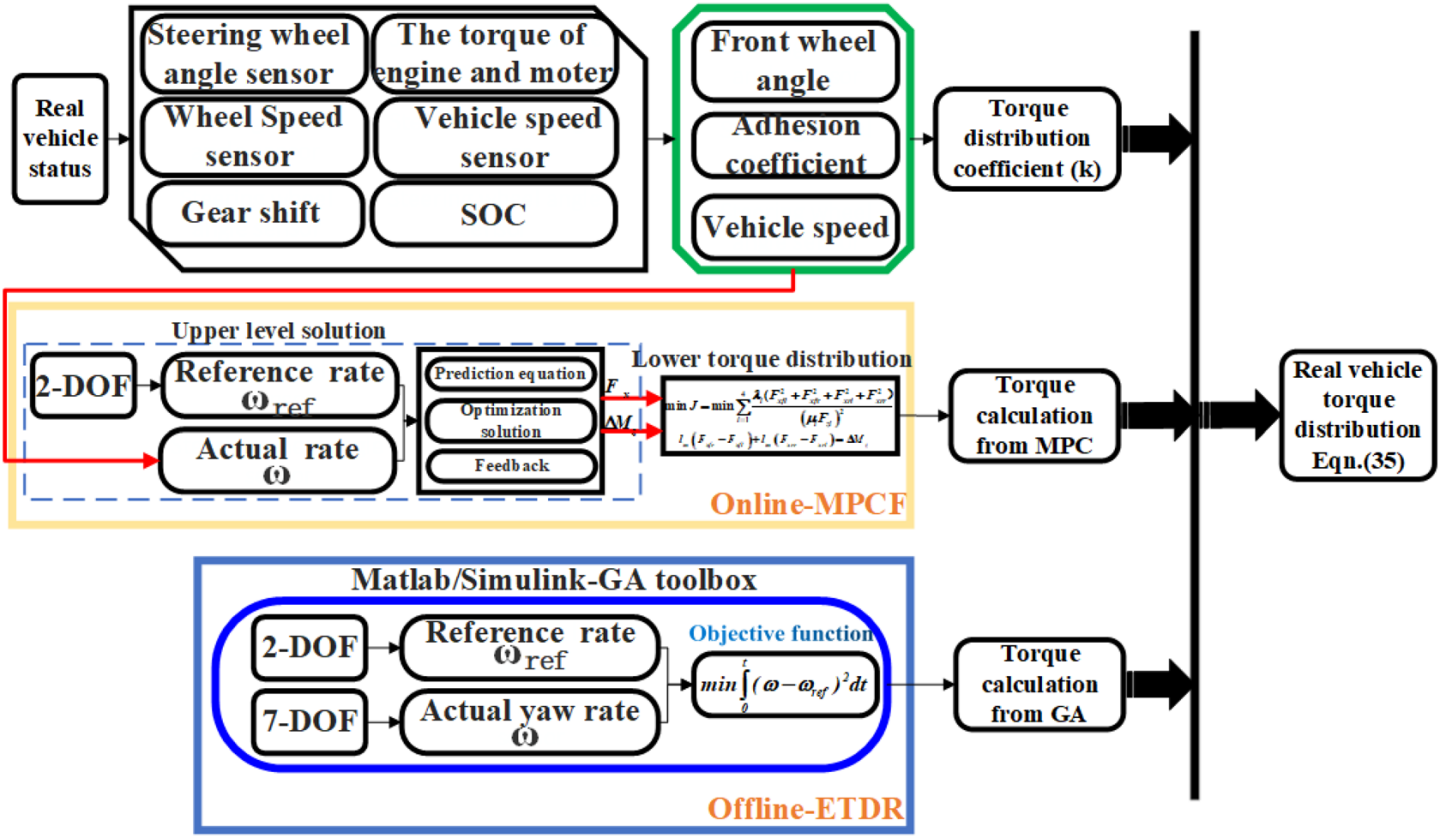
Schematic diagram of stability control strategy.

When the whole vehicle moves in region A (as shown in Table 9), the offline-ETDR can well maintain the motion performance of the whole vehicle. At this time, the wheel torque of the whole vehicle only needs to be distributed according to the offline-ETDR. For other areas, the stability of the whole vehicle cannot be guaranteed only by the results of off-line optimization. Therefore, it is considered to combine offline-ETDR and online-MPCF correction strategy in region B, C and D to improve the handling stability. The design Eq. (35) in this study integrates the wheel torque of the whole vehicle, and the value of *K* is determined by Table 9.

35 $$T_{i} = (1 - k_{i} )T_{offline - ETDR} + k_{i} T_{online - MPCF} \, i = A,B,C,D \,$$

here, the value of *k*_*i*_ in Eq. (35) is determined by Table 9. When the vehicle is in motion in area A, *k*_*A*_ = 0. Similarly, *k*_*B*_ = 0.3, *k*_*C*_ = 0.5, *k*_*D*_ = 0.8 can get. Figure 8 shows the flow of vehicle handling and stability control strategy (HSM) using the combination of offline-ETDR and online-MPCF correction strategy.

## Simulation and experimental verification

### Numerical simulation results

**Figure 9:**
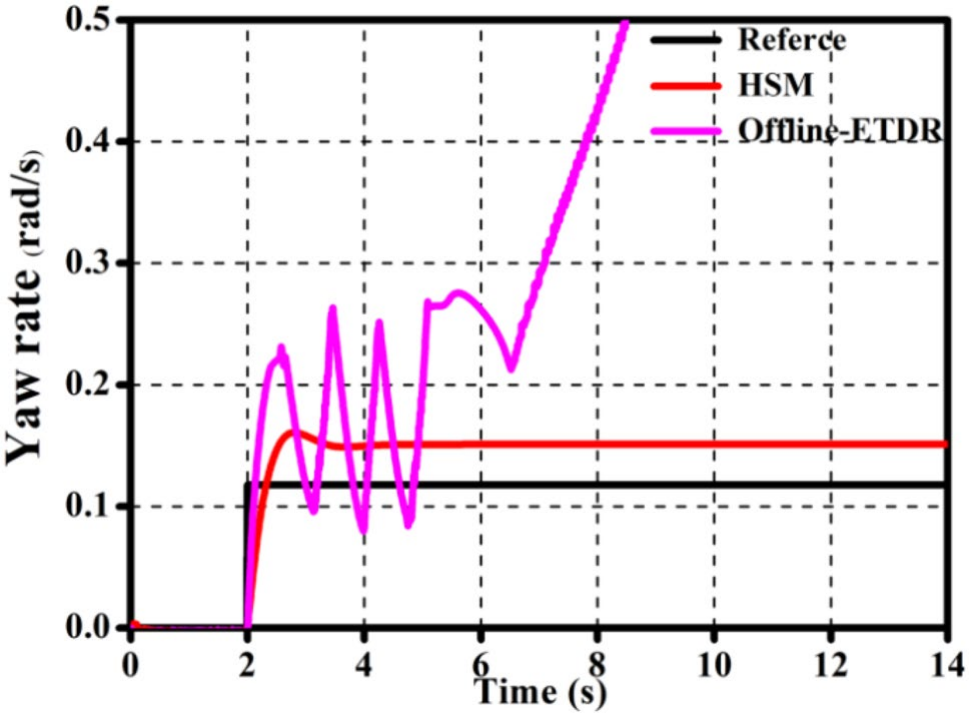
Yaw rate under different methods.

Figure 9 depicts the yaw rate control impact of HSM control strategies at *v* = 80 km/h, *μ* = 0.6, and *θ* = 0.05 rad. Compared with the* ω* under solely offline-ETDR in Fig. 7a, the suggested control approach may bring the *ω* as close to the reference as possible under difficult operating conditions.

The representative step condition, single lane condition and double lane condition were chosen to assess the efficacy of the stability management technique described in this research under varied adhesion coefficient roads. The simulation setup conditions are shown in Table 10.

**Table 10:** Simulation setting conditions.

Condition	Coefficient of adhesion	Front wheel angle (rad)
Step condition	0.3, 0.8	2–14 s: 0.04 rad
Single line	0.3, 0.8	2–6 s: 0.04 rad, 6–10 s: − 0.04 rad
Double line	0.3, 0.8	2–6 s: 0.04 rad, 6–14 s:− 0.04 rad, 14–18 s: 0.04 rad

The front wheel angle is positive, it means turning left. The velocity is 80 km/h.

#### Simulation results under the low adhesion condition

**Figure 10:**
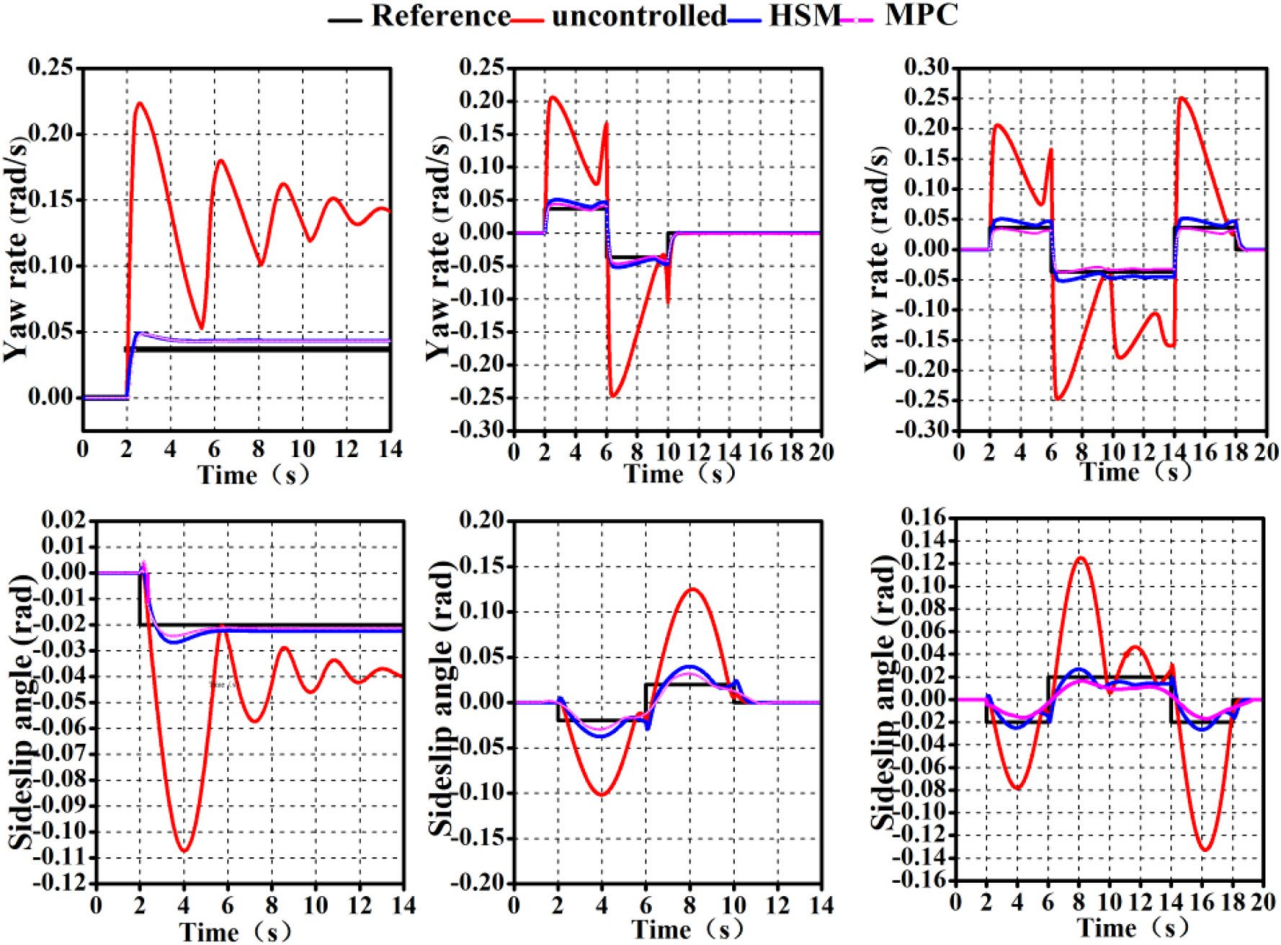
Simulation of low adhesion pavement.

The effectiveness of employing the HSM technique on a poor adhesion road is depicted in Fig. 10. In comparison to the movement of the entire vehicle without control strategy, the HSM can ensure that the* β* and *ω* of the vehicle always follow the reference value, which has improved the overall vehicle stability under extreme working conditions and achieved a suboptimal control effect compared to MPC.

#### Simulation results under the high adhesion condition

**Figure 11:**
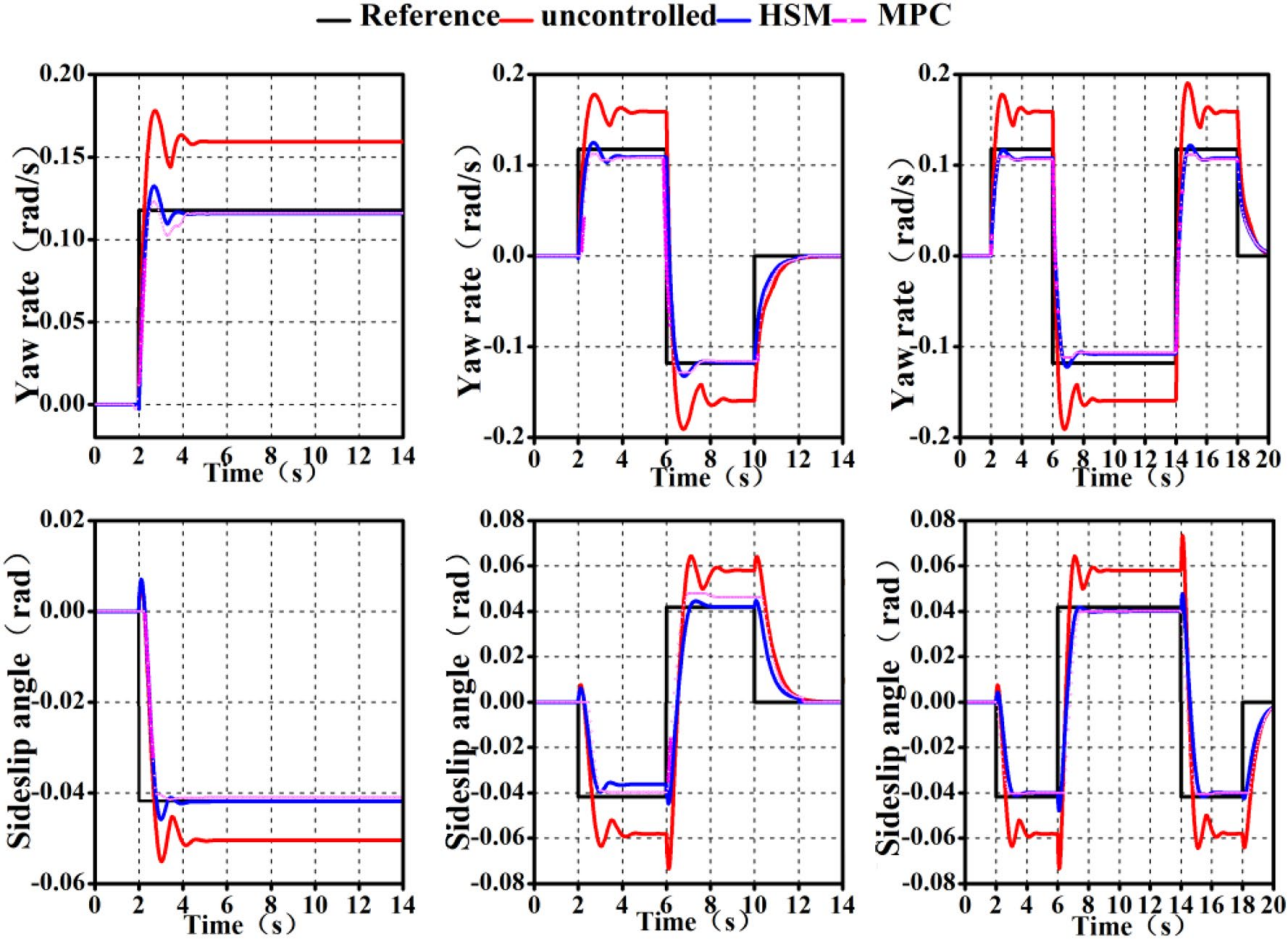
Simulation of high adhesion pavement.

The Fig. 11 shows that the peak values of *β* and *ω* are lower under the condition of a high adhesion road than they would be under a low adhesion road. However, the HSM technique that combines offline-ETDR and online-MPCF also demonstrates clear benefits, namely, the ability of actual values *β* and *ω* to consistently follow the reference value.

### Test verification in the real-world vehicles

**Figure 12:**
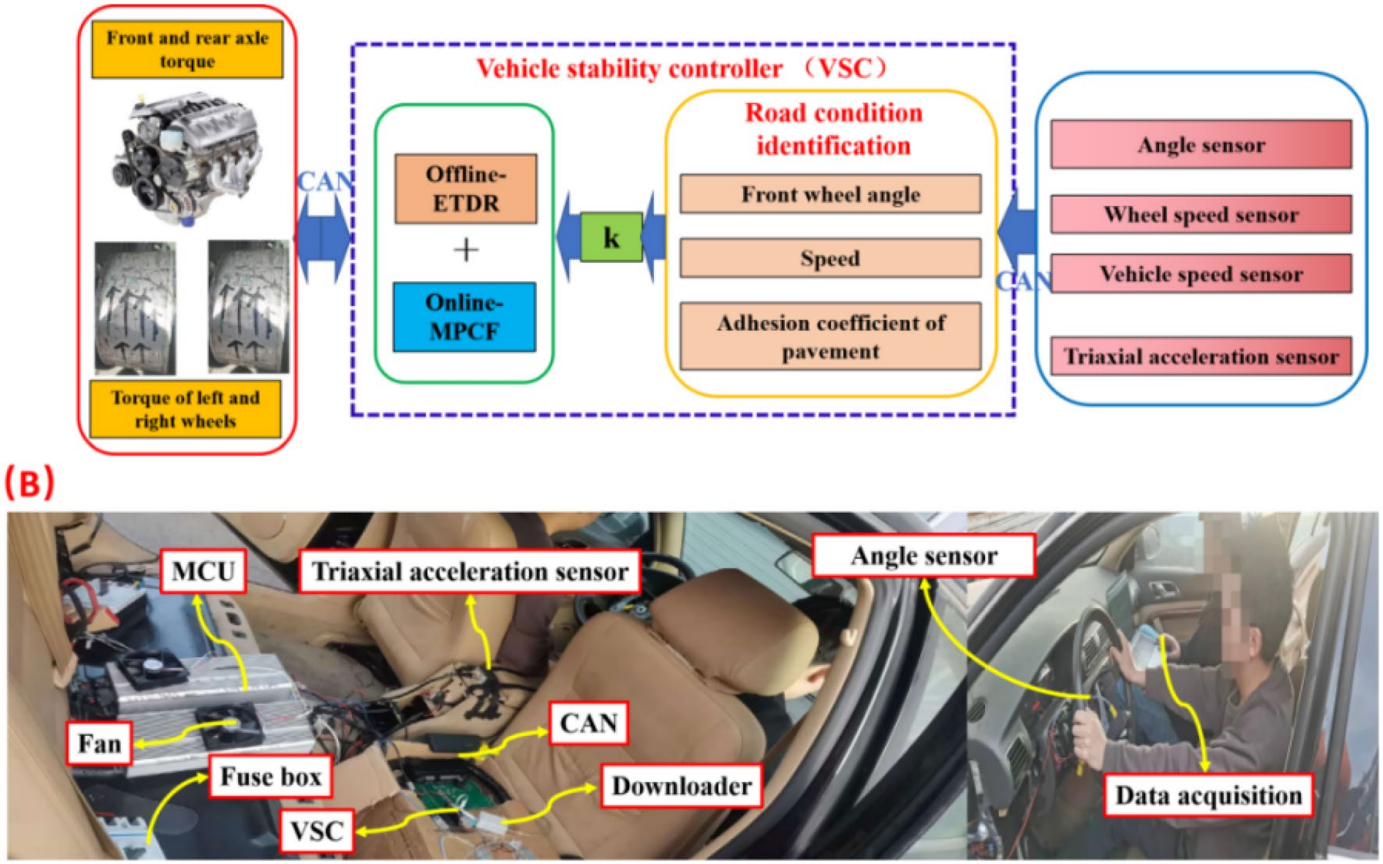
The test platform for TTR.

The stability test platform of the TTR vehicle is established in this research as illustrated in Fig. 12. The angle signal, wheel speed information, yaw angular acceleration, and lateral acceleration signals recorded by the steering wheel angle sensor, wheel speed sensor, and three-axis accelerometer are sampled and transmitted to the vehicle controller through the CAN bus. Following the integrated computation of the vehicle controller, the vehicle speed, front wheel angle, road adhesion coefficient, yaw angular velocity, and side deflection angle are determined, and the driving road conditions are judged based on the current sampled angle signals. Moreover, all experimental protocols were approved by the Beijing Institute of Technology.

According to the division of effective areas for using offline control rules, if the current driving road conditions are suitable for directly using offline control rules, it is sufficient to directly call and use offline-ETDR in the vehicle controller, if the offline-ETDR cannot be directly used in the current driving road conditions, the torque distribution ratio of front and rear axles and left and right wheels can be calculated in real time through the vehicle controller. Equation (35) is used to synthesize the off-line and on-line corrective torques in conjunction with the detection of the present driving road conditions. The vehicle stability controller delivers the fused wheel torque to the motor controller and engine controller through CAN, completing the whole control process.

#### Test results under the single lane condition

**Figure 13:**
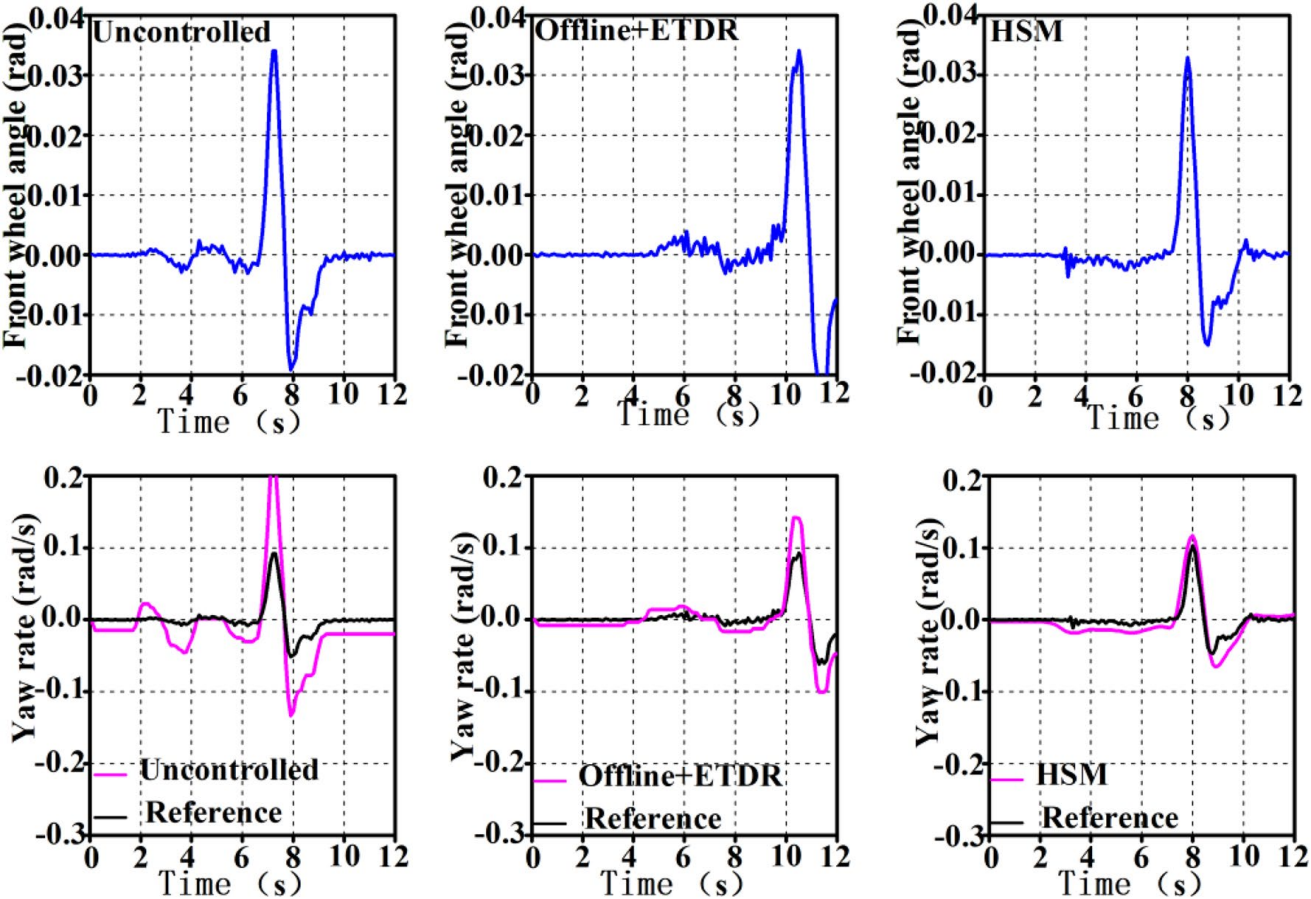
Yaw rate of single lane.

The test velocity is set at 60 km/h, and the single lane test whose standard can be found from GB/T 6323-2014 is performed on a cement pavement with an adhesion coefficient of roughly 0.8. The front wheel angles are somewhat different each time due to the impact of driver manipulation, although they are pretty similar. Figure 13 depicts the test findings and Table 11 counts the peak deviation and area relative deviation of the test value and reference value of *ω* under single lane condition to demonstrate the success of the suggested control technique. The peak deviation indicates the instantaneous steering characteristics of the vehicle, whereas the area relative deviation represents the average steering and driving characteristics of the vehicle.

**Table11:** Peak value deviation and area relative deviation of yaw rate under single lane.

	No control	RB (%)	RB + MPC (%)
dev	57.1%	30.7	18.1
devE	1	24.2	10.3

The peak deviation is defined as follows:

36 $${\text{dev}} = \frac{{\omega_{h} - \omega_{rh} }}{{\omega_{h} }} \times {\text{100\% }}$$

where symbol *dev* is the peak deviation, *ω*_*h*_ is the peak value of the actual yaw rate and *ω*_*rh*_ is the peak value of the reference yaw rate.

Area deviation is defined as follows:

37 $$E_{1} = \int\limits_{0}^{t} {{(}\omega { - }\omega_{r} {) }} dt \, E_{2} = \int\limits_{0}^{t} {{(}\omega { - }\omega_{r} {) }} dt \, E_{3} = \int\limits_{0}^{t} {{(}\omega { - }\omega_{r} {) }} dt$$

Further define the relative deviation of area:

38 $$devE_{1} = \frac{{E_{1} }}{{E_{1} }} \times {\text{100\% }}devE_{2} = \frac{{E_{2} }}{{E_{1} }} \times {\text{100\% }}devE_{3} = \frac{{E_{3} }}{{E_{1} }} \times {\text{100\% }}$$

where *E*_*1*_ is the area deviation without control strategy, *E*_*2*_ is the area deviation of offline control rule strategy, *E*_*3*_ is the area deviation of offline and online combined control strategy, *ω* is the actual yaw rate, *ω*_*r*_ is the reference yaw rate; and *t* is the actual operation time.

Table 11 shows that the peak deviation and area relative deviation between the *ω* of the whole vehicle and the reference are the smallest when the HSM strategy is used, indicating that the HSM strategy optimizes the whole vehicle in the transient and overall driving process. Although the peak deviation and area relative deviation of *ω* under off-line rule control are not as excellent as the overall vehicle motion performance under the HSM strategy, they are better than the control effect without control strategy.

#### Test results under the double lane condition

**Figure 14:**
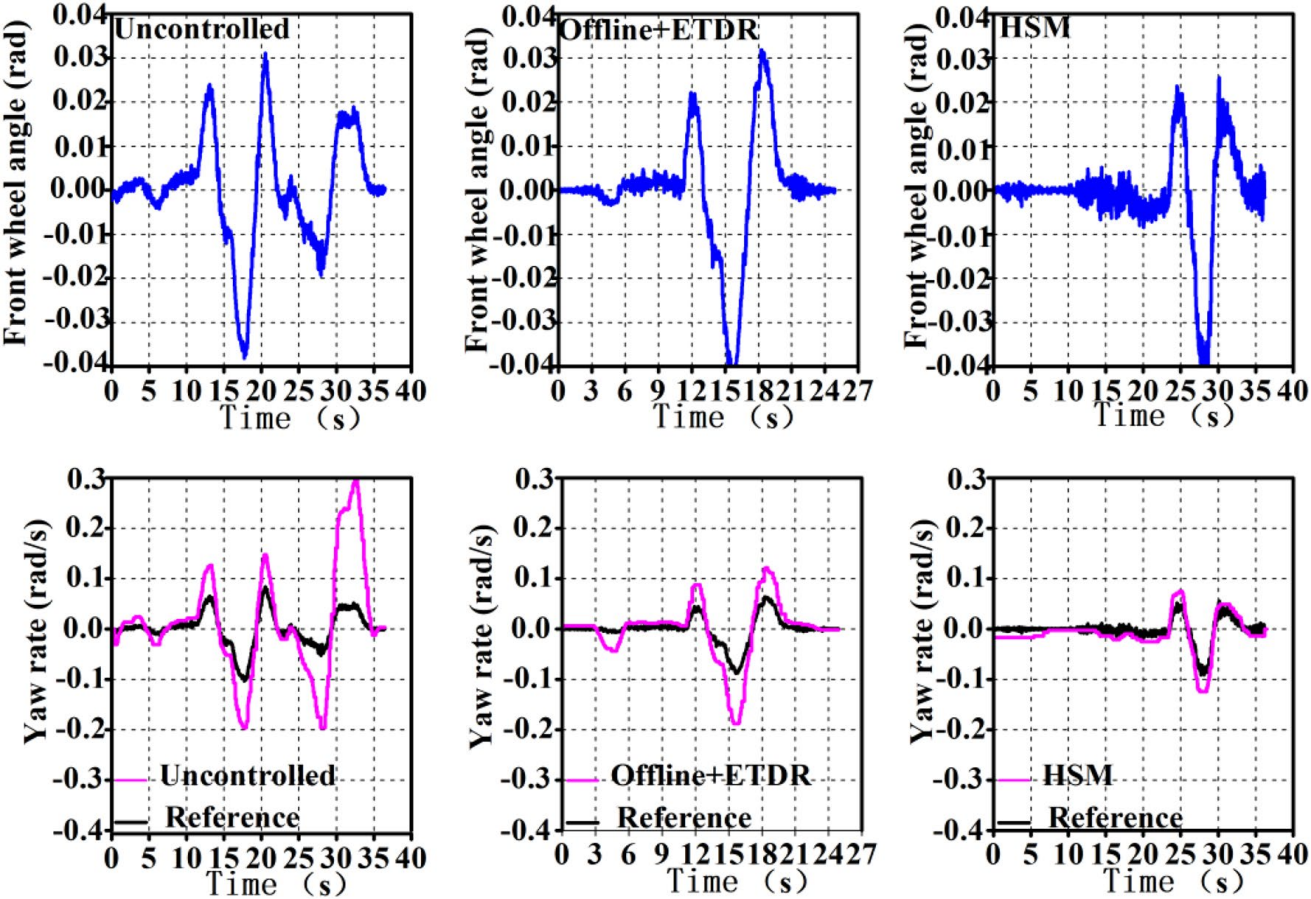
Yaw rate of double lane.

The test velocity is set at 60 km/h, and the stability verification is performed on a cement pavement with a pavement adhesion coefficient of roughly 0.8 during the double lane working situation whose standard can be found from GB/T 6323-2014. Figure 14 depicts the test findings and Table 12 displays the peak deviation and area relative deviation of the test and reference values of the *ω* under the double lane working condition.

**Table12:** Peak value deviation and area relative deviation of yaw rate under double lane.

	No control	RB (%)	RB + MPC (%)
dev	53.8%	40	25
devE	1	28.6	11.4

Table 12 shows that compared to the control effect without a control strategy, the off-line control rule approach and the HSM strategy may lower the peak deviation and area relative deviation of the yaw rate of the whole vehicle. The HSM control strategies can improve the overall motion performance of the vehicle.

## Conclusion

Aiming at the stability problem of TTR 4WD hybrid electric vehicle, this paper develops an integrated stability control strategy based on the offline-ETDR formulation and online-MPCF correction. The simulation and experimental results have demonstrated the following points.

1. A 7-DOF model is used to extract the torque distribution rules offline, and MPC feedback link is introduced to correct the torque distribution online for some regions with the poor results.
2. According to the road condition recognition, the offline-ETDR and the torque corrected by online-MPCF are fused. The simulation results show that the proposed control strategy can significantly improve the vehicle's extreme cornering ability and ensure better handling and stability compared with the vehicle motion without control.
3. Under the single lane and double lane driving situations, the maneuverability and stability approach is tested in the real-world vehicles. Compared with no control, the control strategy of HSM developed in this paper can reduce the peak deviation and area deviation of yaw rate by 18.1% and 10.3% under the single lane condition, and 25% and 11.4% under the double lane shifting condition. The test results demonstrate that the HSM technique put forth in this study has enhanced the overall vehicle's handling stability.
4. For safety reasons, this paper only conducts a preliminary functional verification of stability control under low speed and high adhesion road conditions through experiments. The verification of driving conditions on extreme roads is the urgent content. In addition, further design of the state observer for the sideslip is also the future research direction.

## Data Availability

The datasets used and/or analysed during the current study available from the corresponding author on reasonable request.

## Abbreviations

TTR: Through-the-road
4WD: 4-Wheel-drive
HSM: Handling and stability management
MPC: Model predictive control
Offline-ETDR: Offline extract torque distribution rules
Online-MPCF: Online MPC feedback
AS: Active suspension
ESP: Electronic stability program
ASC: Active steering control
SOC: State of the charge
AMT: AutoManual transmission
RB: Rule based control
DYC: Direct yaw control
PID: Proportion-integral-derivative
SMC: Sliding mode control
RL: Reinforcement learning
7-DOF: Seven degrees of freedom
2-DOF: Two degrees of freedom

## Symbols

F_x_: The total longitudinal force
F_xfl_: The longitudinal forces of the left front wheel
F_xf__r_: The longitudinal forces of the right front wheel
F_x__r__l_: The longitudinal forces of the left rear wheel
F_x__rr_: The longitudinal forces of the right rear wheel
F_y_: The total lateral force
F_yfl_: The lateral forces of the left front wheel
F_yf__r_: The lateral forces of the right front wheel
F_y__r__l_: The lateral forces of the left rear wheel
F_y__rr_: The lateral forces of the right rear wheel
M_z_: The total yaw moment
∆M_z_: The additional yaw moment
a: The distance from the front axle to the center
b: The distance from the rear axle to the center
y: The output variable
ω: The yaw rate
T: The sampling time
Q: The weight coefficient
v: Vehicle speed
l_m_: The half the track width
θ: The front wheel angle
C_D_: The air resistance coefficient
A: The windward area
Td: The driving torque of the whole vehicle
r: The tire radius
T_e_: The torque of the engine
Tm: The torque of the hub motors
$\upeta$: The transmission efficiency
i_0_: The speed ratio of the main reducer
i_g_: The speed ratio of each gear of the gearbox
x: The state variable
u: Control variable
β: The sideslip angle
m: Vehicle weight
λ_i_: Constant
μ: Road adhesion coefficient
F_zi_: The vertical force of each wheel
α_f_: Tire sideslip angle
